# Durable Response to Lenvatinib in Platinum-Refractory Metastatic High-Grade Thymic Mucoepidermoid Carcinoma: A Case Report

**DOI:** 10.1016/j.jtocrr.2025.100894

**Published:** 2025-08-22

**Authors:** Noa Amin, Thanika Ketpueak, Simon Jordan, Andrew G. Nicholson, Yu Zhi Zhang, Sanjay Popat

**Affiliations:** a Lung Unit, Royal Marsden Hospital, London, United Kingdom; b Faculty of Medicine, Chiang Mai University, Chiang Mai, Thailand; c Department of Thoracic Surgery, Royal Brompton and Harefield NHS Hospitals, Guy’s and St Thomas’ NHS Foundation Trust, London, United Kingdom; d Department of Histopathology, Royal Brompton and Harefield NHS Hospitals, Guy’s and St Thomas’ NHS Foundation Trust, London, United Kingdom; e National Heart and Lung institute, Imperial College London, United Kingdom; f Institute of Cancer Research, London, United Kingdom

**Keywords:** Case report, Lenvatinib, MAML2 fusion, Mucoepidermoid carcinoma, Thymic carcinoma

## Abstract

Thymic mucoepidermoid carcinoma (MEC) is a rare thymic carcinoma subtype. Current metastatic thymic carcinoma guidelines recommend first-line platinum-based chemotherapy. However, evidence suggests that MECs, including those of the lung and salivary gland, are chemorefractory, highlighting a more nuanced approach to systemic therapy decision-making. Here, we report a case of durable partial response to second-line lenvatinib in a patient with metastatic high-grade thymic MEC, refractory to first-line platinum-based chemotherapy, suggesting a potentially preferred first-line role for lenvatinib for this subtype.

## Introduction

Mucoepidermoid carcinoma (MEC) is the most common malignant tumor subtype of the salivary gland, but it can also occur at other sites, including the thymus, albeit very rarely, accounting for approximately 2% of thymic tumors. It is currently classified under salivary glandlike carcinomas of the thymus in the 2021 WHO thoracic tumors classification^1^ and is characterized by a CRTC1::MAML2 fusion.^2^ High tumor grade confers a poor prognosis in thymic MEC,^3^^,^^4^ and optimal systemic therapy remains poorly characterized, with no histology-specific preferred systemic therapies for thymic carcinoma subtypes. Herein, we report a case of metastatic high-grade thymic MEC, platinum-refractory, but with a durable response to lenvatinib.

## Case presentation

A 47-year-old male with unremarkable past medical history presented with persistent hiccups and exertional dyspnea in November 2022. Imaging revealed an anterior mediastinal mass, with lung, liver, pancreatic, soft tissue, bone, nodal metastases, pericardial, and pleural effusions. Mediastinal mass biopsy identified a poorly differentiated carcinoma. Biomarker analysis identified a programmed death-ligand 1 tumor propensity score of 2% and in-house tissue DNA and RNA next-generation sequencing only identified a somatic *BAP1* deletion and a *CRTC1*::*MAML2* fusion.

**Figure 1:**
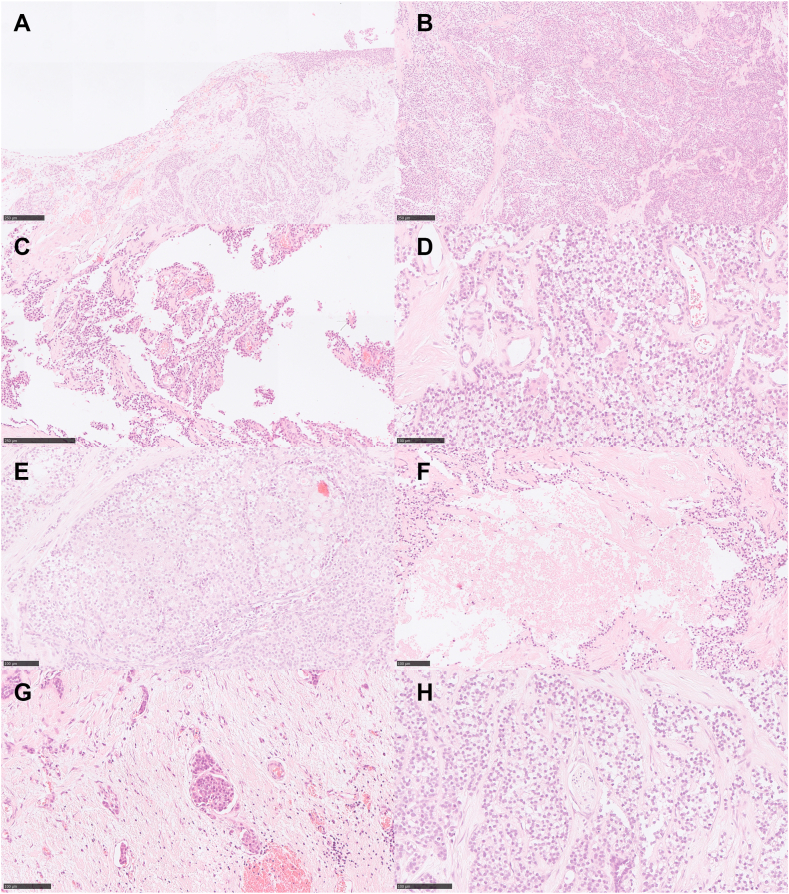
Histopathologic Features. (*A*) The tumor invades the mediastinal pleura (right) with surface tumor deposits. (*B*) Most of the tumor exhibited a solid growth pattern. (*C*) Focal papillary growth pattern. (*D*) The predominant tumor cell population are intermediate cells with clear cell morphology, intermixed with a minor population of epidermoid cells containing abundant eosinophilic cytoplasm resembling squamous epithelial cells. (*E*) Mucous cells, characterized by those containing cytoplasmic vacuoles with pale basophilic mucin, are rarely observed. (*F*) Tumor necrosis. (*G*) Lymphovascular invasion. (*H*) Perineural invasion (center).

A diagnosis of T2N1M1b metastatic high-grade thymic MEC was made, and he commenced carboplatin-paclitaxel. After one cycle, he developed a significant left-sided pleural effusion and subsequently underwent video-assisted thoracoscopic surgery for pleural drainage. Pleural biopsies again confirmed MEC, with similar characteristics but also high-grade histologic features, including solid growth pattern, focal necrosis, lymphovascular and perineural invasion (Fig. 1). Further intervention with talc pleurodesis and phrenic nerve transection was required for progressive hiccups refractory to medical therapy. He continued with two additional cycles of carboplatin-paclitaxel.

**Figure 2:**
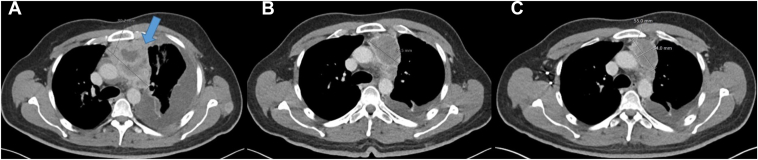
Radiologic results before and after initiation of Lenvatinib. (*A*) Contrast-enhanced CT scan of the thorax revealing a large, heterogeneous anterior mediastinal mass, denoted by arrow, before initiation of lenvatinib (baseline CT May 2023). (*B*) Contrast enhanced CT scan revealing PR 2 months post initiation of lenvatinib (July 2023). (*C*) Contrast enhanced CT scan revealing ongoing response to lenvatinib (September 2023). CT, computed tomography, PR, partial response.

In May 2023, after three cycles of carboplatin-paclitaxel, contrast-enhanced imaging revealed progressive disease by virtue of increasing liver, soft tissue, and bony disease and increasing pleural and pericardial effusions (Fig. 2*A*). Incipient cardiac tamponade necessitated emergency pericardial window, indwelling pleural catheter insertion, and peritoneal shunt procedure. Biopsies of the pericardium and diaphragm reconfirmed MEC. His Eastern Cooperative Oncology Group performance status was 3.

He commenced lenvatinib from May 2023 at 20 mg daily with reviews of tolerability and response as per institutional protocol. His clinical condition rapidly improved over 4 weeks with resolution of pain, anorexia, fatigue, and hiccups, and Eastern Cooperative Oncology Group performance status improved to 1. Response-evaluation computed tomography (CT) in July 2023 revealed an excellent partial response (PR) at all sites (Fig. 2*B*). Subsequent CT in September 2023, post four cycles of lenvatinib, confirmed ongoing PR (Fig. 2*C*). Apart from intermittent grade 1 nausea, papulopustular rash, and fatigue, he tolerated lenvatinib well without significant toxicities. He did not have proteinuria or elevated blood pressure.

**Figure 3:**
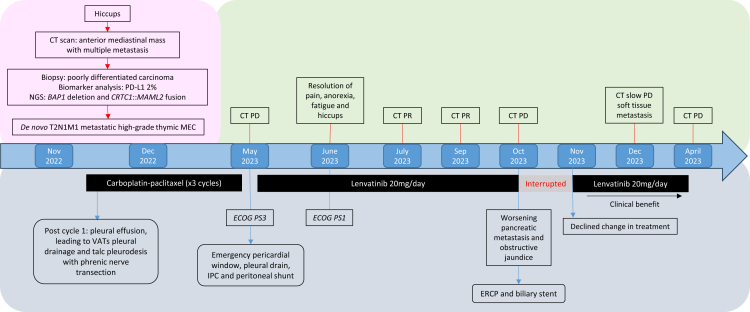
Timeline of events and clinical course. CT, computed tomography; EGOG PS, eastern cooperative oncology group performance status; ERCP, endoscopic retrograde cholangiopancreatography; MEC, mucoepidermoid carcinoma; VATs, video-assisted thoracoscopic surgery.

In October 2023, he developed obstructive jaundice because of a marginally deteriorating pancreatic lesion on CT. Magnetic resonance cholangiopancreatography confirmed biliary duct obstruction secondary to a pancreatic head metastasis, and he underwent a successful sphincterotomy and biliary stent placement during endoscopic retrograde cholangiopancreatography. There was no withdrawal of lenvatinib except during this period of obstructive jaundice. After discussion and declining a change in further treatment, he continued lenvatinib in December 2023, but with slowly enlarging soft tissue metastases, continuing with clinical benefit until April 2024, when he developed radiologic progressive disease and tumor fistulation. He was symptomatically managed and discharged with community palliative care support and died in June 2024. For a summary of the timeline of events and clinical course, please refer to Figure 3.

## Discussion

MEC, initially described as a distinct histologic subtype of salivary gland tumors, is also reported in the respiratory tract, esophagus, breast, and thymus. Platinum-based therapy, such as carboplatin-paclitaxel, is guideline-recommended as first-line for metastatic thymic carcinoma. The rarity of metastatic thymic MEC has led to limited systemic therapy data for this subtype.

We described a high-grade thymic MEC with a *CRTC1*::*MAML2* fusion and chemorefractoriness as observed in MECs from salivary gland or lung, highlighting an unmet need for efficacious pharmacotherapy for this rare subtype. We reported a durable response to lenvatinib, underpinning a symptomatic improvement. Lenvatinib is an antiangiogenic multitargeted kinase inhibitor of VEGFR1-3, FGFR1-4, PDGFR, RET, c-kit, and PDGFRα. High expression of VEGF receptors has been reported in thymic epithelial malignancies. Whereas lenvatinib has exhibited activity in two phase II trials for thymic carcinoma pretreated with platinum-based chemotherapy and is licensed for this indication in Japan, its efficacy by histologic subtype remains unclear. The Japanese phase II REMORA study evaluated lenvatinib in thymic carcinomas, reporting an objective response rate (ORR) of 38%, a median progression-free survival (PFS) of 9.3 months, and a median overall survival (OS) of 28.3 months.^5^ The phase II PECATI trial of lenvatinib-pembrolizumab combination in advanced B3 thymoma or thymic carcinoma reported an ORR of 23% with a median PFS of 14.9 months.^6^ In addition, in vivo studies suggest sensitivity to inhibition of EGFR and cell cycle signaling in CRTC1::MAML2-positive MEC.^7^ Our case is supported by data from two phase II studies in advanced salivary adenoid cystic carcinoma reporting an ORR of 11% to 16% and PFS of 9.1 to 17.5 months for lenvatinib monotherapy.^8^^,^^9^

The patient remained on lenvatinib for approximately 12 months with maintained and clinically meaningful PR as the best overall response. Although it is important to consider the potential treatment-related adverse events of lenvatinib, and that the generalizability of any individual case necessitates interpretation with caution, our case supports the prioritization of lenvatinib over chemotherapy in advanced thymic MEC.

In conclusion, we report a case of metastatic high-grade thymic MEC with a CRTC1::MAML2 fusion, refractory to guidelines-based first-line platinum-based chemotherapy but with a durable and marked clinical response to lenvatanib. This case highlights lenvatinib as an efficacious treatment choice for advanced thymic MEC and supports its prioritization over chemotherapy.

## CRediT Authorship Contribution Statement

**Noa Amin:** Writing - original draft.

**Thanika Ketpueak:** Writing - original draft.

**Simon Jordan:** Writing - review & editing.

**Andrew G. Nicholson:** Writing - review & editing.

**Yu Zhi Zhang:** Writing - review and editing.

**Sanjay Popat:** Conceptualization, Supervision, Writing - review & editing.

## Disclosure

Dr. Popat reports receiving personal fees for advisory board participation from Amgen, AnHeart, Arcus Biosciences, AstraZeneca, Bayer, Bicycle Therapeutics, Biontech Rna Pharmacuticals GMBH, BMS, Boehringer Ingelheim, Daiichi Sankyo, Eisai, Ellipses, Erasca, Genmab, Gilead, GSK, Guardant Health, IO Biotech, Janssen, Lilly, MSD, Novartis, Pfizer, Pierre Fabre, Regeneron, Taiho and Takeda; personal fees as an invited speaker from Medscape, Novocure, and Pharmamar; personal fees for expert testimony from Merck Serono and Roche; institutional fees as a subinvestigator for Amgen and MSD; institutional fees as a local PI for AstraZeneca, GSK, and Roche; institutional fees as a coordinating principal investigator (PI) for BMS, Boehringer Ingelheim, Daiichi Sankyo, Janssen, Lilly, Roche, Takeda, and Turning Point Therapeutics; institutional research grant from 10.13039/100031949Guardant Health; nonremunerated advisory roles for ALK-Positive United Kingdom, International Association for the Study of Lung Cancer (IASLC), Lung Cancer Europe, ROS1dersUK, and Ruth Strauss Foundation; nonremunerated leadership roles for the British Thoracic Oncology Group (Chair of the Research Committee) and the European Thoracic Oncology Platform (Foundation Council Member); and nonremunerated member of the Thoracic Faculty for European Society For Medical Oncology (ESMO). The remaining authors declare no conflict of interest.
